# Ruptured Endometrioma Associated With Acute Appendicitis: A Case Report

**DOI:** 10.7759/cureus.76136

**Published:** 2024-12-21

**Authors:** Abdelhamid Maqsoudi, Hamza Mougib, Ouazni Mohamed, Mehdi Soufi, Soukaina Wakrim

**Affiliations:** 1 Department of Radiology, Souss Massa University Hospital Center, Faculty of Medicine and Pharmacy, Ibn Zohr University, Agadir, MAR; 2 Department of General Surgery, Souss Massa University Hospital Center, Faculty of Medicine and Pharmacy, Ibn Zohr University, Agadir, MAR

**Keywords:** abdominal imaging, acute appendicitis, case report, computed tomography, emergency radiology, hemoperitoneum, pelvic pain, ruptured endometrioma, ultrasonography

## Abstract

Endometrioma is a localized form of endometriosis, usually found within the ovaries bilaterally, containing degenerated blood products resulting from bleeding of ectopic endometriotic tissue at different ages. Rupture of the endometrioma is a rare complication that may result in hemoperitoneum and peritonitis and thus presents similarly to other more common abdominal emergencies, and the concomitant presence of a ruptured endometrioma and another abdominal emergency, although exceptional, remains possible. Ultrasonography and sectional imaging can be used to assess a diagnosis that is often confirmed postoperatively. We present a case of a ruptured endometrioma with diffuse suppurated hemoperitoneum associated with acute appendicitis that was initially diagnosed as a complicated tubo-ovarian abscess.

## Introduction

Endometrioma rupture, although rare, represents a significant diagnostic challenge in emergency settings due to its ability to mimic other acute abdominal and gynecological conditions, such as ruptured ovarian cysts, ectopic pregnancy, or pelvic inflammatory disease. The clinical presentation often includes acute onset of lower abdominal pain, which may be accompanied by nausea, vomiting, or vaginal bleeding, complicating the differentiation from other pathologies.

The reported incidence of ruptured endometriomas is low, occurring in approximately 2-3% of cases, but when it does occur, it can lead to peritoneal irritation, hemoperitoneum, or even sepsis in severe cases [1,2]. Moreover, the coexistence of a ruptured endometrioma and another surgical emergency, such as appendicitis, though exceedingly uncommon, has been documented in case reports, underscoring the complexity of these scenarios [3].

Imaging modalities, particularly ultrasound and computed tomography (CT), play a crucial role in the initial evaluation. Ultrasound may reveal an adnexal mass with echogenic fluid suggesting hemorrhage, while CT can help delineate associated complications such as hemoperitoneum or secondary inflammation [4,5]. Despite these advances in diagnostic imaging, surgical exploration, often via laparoscopy, remains the gold standard for establishing a definitive diagnosis and addressing associated complications [6].

## Case presentation

**Figure 1:**
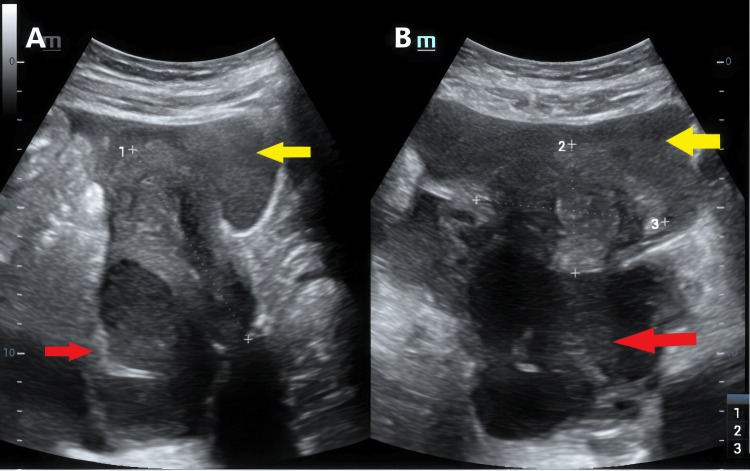
Ultrasound (sagittal and axial views) showing a retro-uterine, multi-loculated, thick-walled cystic structure with diffuse low-level internal echoes (red arrow) and intraperitoneal echoic free fluid (yellow arrow).

A 24-year-old woman presented to the emergency department with acute pelvic pain that had abruptly started for less than 24 hours and increased in intensity. Upon examination, the patient had mild fever and diffuse abdominal tenderness that was more intense in the right iliac fossa with muscle guarding. The patient gave a history of being a virgin and never having any sexual intercourse; she had no late period but reported a mild dark uterine discharge that day. The patient had a history of chronic pelvic discomfort. The complete blood count found an elevated white cell count of 27570 cells/µL (the reference range for adults is 4,000-11,000 cells/µL), and C-reactive protein was also elevated at 215.9 mg/L (normal: <5 mg/L). Human chorionic gonadotropin (HCG) was negative. An acute appendicitis was suspected and an ultrasound was performed and objectified a retro-uterine, multi-loculated, thick-walled cystic structure with diffuse low-level internal echoes, and a solid component with no Doppler signal. It extended to both adnexal regions and seemed to be abutting an ovary found on the right, recognized by its follicles with no clear distinction between the ovary and its tube (Figure 1). Diffuse and abundant intraperitoneal echoic free fluid was also found, but the appendix could not be visualized.

**Figure 2:**
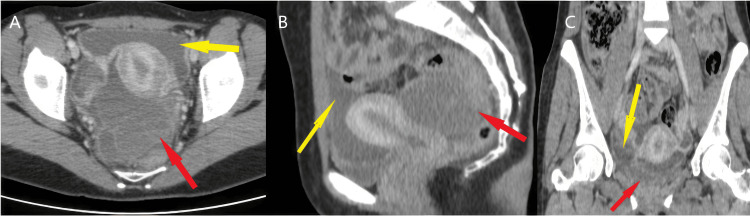
Enhanced CT scan (axial (A), sagittal (B), and coronal (C) views) showing a multi-loculated, retro-uterine, thick-walled, slightly enhancing cyst (red arrow) with intraperitoneal fluid (yellow arrow).

In light of these clinical, biological, and radiological findings, the diagnosis of acute peritonitis with a right tubo-ovarian abscess as a starting point complicating a pelvic inflammatory disease was proposed, and CT was performed for further characterization given the unavailability of MRI. The CT objectified the same retro-uterine, multi-loculated cystic mass measuring approximately 110 × 100 mm (Figure 2), with a thick wall slightly enhancing after injection of contrast product that seemed to be immerging from the left annex without a clear distinction between the left ovary and its tube, since the right ovary could not be visualized.

**Figure 3:**
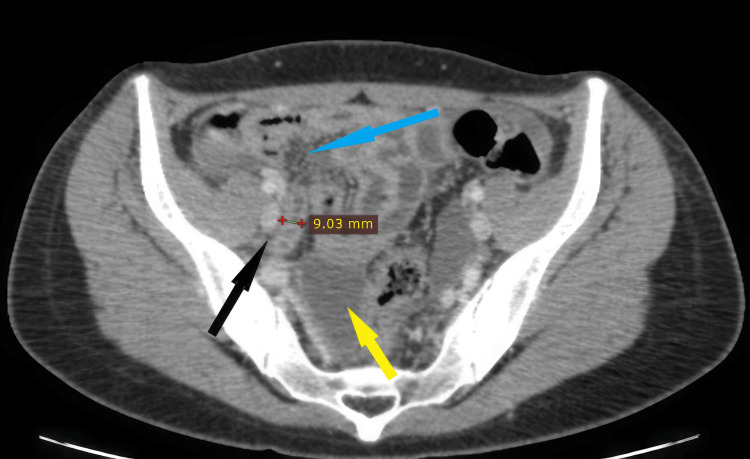
Enhanced CT scan (axial view) showing a thickened appendix (black arrow) with an infiltrated mesenteric fat (blue arrow) and free fluid (yellow arrow).

The CT also demonstrated a diffuse intraperitoneal liquid with regular and diffuse peritoneal thickening and a thickened appendix measuring 9 mm with an infiltrated appearance of the mesenteric fat (Figure 3). The diagnosis of acute generalized peritonitis complicating a left tubo-ovarian abscess associated with acute appendicitis was retained.

**Figure 4:**
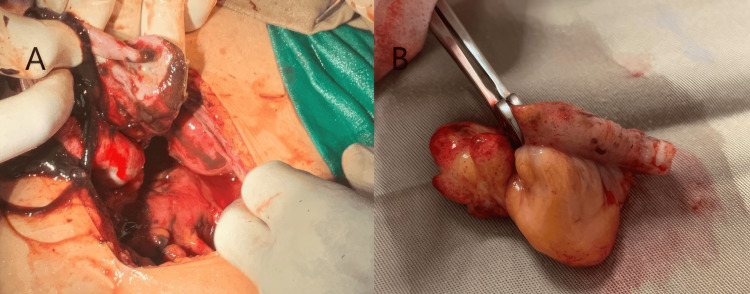
(A) Perioperative picture of the ruptured endometrioma of the left ovary with the hemoperitoneum. (B) Inflamed appendix post appendectomy.

Due to the unavailability of laparoscopy, the patient underwent a laparotomy, which revealed a ruptured left endometrioma, accompanied by brown-chocolate-free intraperitoneal fluid and a thickened appendix. No other endometriotic implants were observed in the abdominal cavity, and no additional endometriotic cysts were noted on either ovary. A cystectomy with ovarian preservation and an appendectomy were performed (Figure 4). Pathological examination confirmed a hemoperitoneum secondary to the ruptured endometrioma, with no evidence of malignancy, and a suppurated appendix.

## Discussion

Endometriomas, also known as endometriotic cysts, are a localized form of endometriosis and consist of thick-walled cysts containing dark and dense degenerated blood products from the recurrent hemorrhage of ectopic endometrial tissue [5]. They are usually found within the ovary and are bilateral in approximately 50% of the cases [7]. They account for 17-44% of women diagnosed with endometriosis [8]. The rupture of endometrioma is rare, with an estimated incidence of less than 3% among women of reproductive age affected by endometriomas [1]. Few case reports are scattered in the literature with some cases associated with pregnancy [9,10].

Size seems to be the most predictive factor of rupture, and the larger the cyst (>6 cm), the greater the risk of rupture [2,11], which explains the occurrence of this situation more frequently in pregnancy in which cysts tend to grow in size due to hormonal stimulation of the endometrial epithelium, stroma, and glands, and may undergo decidualization and become fragile [6,10,11]. However, two studies found that most endometriomas, especially cysts <6 cm, tend to decrease in size [11,12]. Pregnancy also increases tension on the cyst when the uterus increases in size, and alters the anatomical position of the ovary. Other factors that may favor the rupture of endometriomas are trauma, adhesions, and infection. Trauma puts pressure on the cyst wall, and changes in position cause spontaneous rupture in cases of dense endometriosis-associated adhesion [11]. Infection may weaken the endometrioma wall and thus favor its rupture; we believe that this was the case in our patient. The close proximity of the cyst to the infected appendix suggests that bacterial translocation from the appendix was the most likely cause of the cyst's infection and subsequent rupture, rather than the reverse. This conclusion is supported by histopathological findings, which identified lymphoid hyperplasia as the cause of the appendicitis, establishing it as the primary site of infection. Furthermore, the laparotomy revealed no endometriosis-associated adhesions, ruling out any dynamic factors contributing to the rupture.

Most patients who have experienced endometriotic cyst rupture present with sudden onset abdominal pain, nausea, and vomiting, and can identify the timing because of maximal pain and symptom onset [2]. Subsequently, they may develop severe peritonitis and systemic disturbances [2,8].

Endometrioma rupture may result in the spreading of endometriotic fluid into the pelvic cavity, which may induce severe peritonitis and further endometriotic-associated adhesions [8]; thus, the diagnosis should be considered differential in women presenting with sudden abdominal pain, especially those with known endometrioma or a history of dysmenorrhea or chronic pelvic pain because emergent surgical intervention may lead to a better outcome in terms of prevention of adhesion formation and preservation of fertility by reducing the effect of the endometriotic fluid [2,9].

Imaging may help to assess the diagnosis. Cysts with diffuse low-level internal echoes on ultrasound are a characteristic appearance of endometrioma [4], and the presence of free fluid should raise the diagnosis of rupture [5], especially in emergency studies in patients with a known history of endometrioma. MRI is the gold standard for the exploration of female pelvic pathology. It has high specificity for identifying endometriomas, which are characterized by high signal intensity on T1-weighted images and low signal intensity on T2-weighted images, because of the high concentration of protein and iron within the endometrioma from recurrent hemorrhage (the shading sign) [7,13,14]. Rupture can also be diagnosed with great confidence on MRI by the detection of fluid levels in the pelvic space, although it is an uncommon feature due to varied signal intensity on T1- and T2-weighted images [13]. Due to the unavailability of MRI in an emergency setting and clinical similarities between ruptured endometrioma and other more prevalent abdominal emergencies, CT is frequently performed. It should be noted that atypical presentations, such as heterogeneous multi-loculated cysts, could be interpreted as pelvic inflammatory disease or tubo-ovarian abscess, especially in the presence of fever and high white blood cells [15]. A flabby or depressed surface of the cystic lesion has been reported to be a representative feature of ovarian rupture in studies using MRI and CT [13].

Ruptured endometriomas are rarely diagnosed preoperatively, as they share clinical and radiological similarities with other abdominal emergencies. Surgery remains the gold standard for both etiological treatment and diagnosis affirmation [6].

## Conclusions

In summary, we report a rare case of ruptured endometrioma associated with acute appendicitis, which we believe to be a catalyst event. Imaging plays an important role in assessing the diagnosis; however, in most cases, the diagnosis is established postoperatively. Good communication between the radiologist and the surgeon is essential for determining the suitable time of intervention to reduce mortality (due to sepsis and shock) and morbidity (reduced fertility, adhesion, and pelvic pain) of this pathology.
